# Community Pharmacy Needle Exchange Programme: What Can Analysis of the Data Tell Us about the Changing Drug Market in Ireland?

**DOI:** 10.3390/ijerph21030289

**Published:** 2024-03-01

**Authors:** David S. Evans, Norma Harnedy, Eamon Keenan

**Affiliations:** 1 National Social Inclusion Office, Health Service Executive, D20 KH63 Dublin, Ireland; eamon.keenan@hse.ie; 2 HSE Addiction Services, Health Service Executive, V94 PV34 Limerick, Ireland; normam.harnedy@hse.ie; 3 Department of Public Health and Primary Care, Trinity College Dublin, D24 DH74 Dublin, Ireland

**Keywords:** needle exchange, harm reduction, substance use, Ireland

## Abstract

Community Pharmacy Needle Exchanges are a harm reduction measure that have been established in a number of countries to provide access to sterile injecting equipment for people who inject drugs (PWID). To ensure that they are meeting needs, it is important to monitor the use of the services. This study aimed to determine patterns of needle distribution and return in community pharmacies in Ireland over time. The number of pharmacies, needle packs, clean needles and returned packs was obtained from the Health Service Executive (HSE) Planning and Business Information Unit (PBI). Yearly totals were calculated to show patterns from 2015 to 2022. There has been an 18% decline in the number of pharmacies providing the service since 2015, with a 19% decline in the number of packs provided and a 21% decline in the number of packs returned. The proportion of packs returned was 23% in 2015 and 18% in 2022. There has been a 16% decline in the number of sterile needles provided and a 6% reduction in the average number of needles per individual since 2017. Declining needle use and low rates of used needle return (against a backdrop of large numbers of PWID that have not significantly reduced over time) suggest that there is a need to investigate if community pharmacies in Ireland have the scope to improve their harm reduction impact. This raises questions in terms of the need to both improve and adapt the service against a backdrop of changing drug markets. Key recommendations include the need to review the harm reduction services employed by participating pharmacies when providing new equipment and organising the return of used equipment.

## 1. Introduction

The harm reduction approach underpins drug and alcohol policy in many countries worldwide [1,2,3]. In 2022, 105 countries included harm reduction in their national policies [4]. Harm reduction is a proven public health response aimed at reducing the health and social harms associated with a range of risk activities. It has been included in most definitions of a public health approach to substance use in the last decade [5]. In terms of substance use, this approach focuses on interventions such as needle and syringe exchange programmes, opioid agonist treatment (OAT), overdose antidote provision (naloxone), safer injecting facilities and outreach/education programmes [6]. These aim to reduce the negative consequence of drug use (e.g., overdose and infectious disease transmission from injection drug use), as opposed to stopping drug use itself [7].

HIV prevalence among people who inject drugs (PWID) is approximately seven times greater than the adult population [8]. In addition, worldwide it is estimated that 53% of PWID are infected with hepatitis C [9]. Needle and syringe exchange programmes are a harm reduction measure, which aim to reduce the transmission of HIV and blood-borne viruses among PWID and prevent the sharing of contaminated equipment. A number of reviews have demonstrated the effectiveness of needle and syringe exchange programmes in terms of preventing HIV and other infectious diseases, increasing access to treatment and increasing the likelihood of users reducing or stopping drug use [10,11]. They were first established in Ireland in 1989, when five fixed-site services were established in the Dublin region [12]. These services provide access to sterile injecting equipment and a safe way to dispose of used equipment. They are also used to provide onward referral for disease testing, treatment services and other harm reduction interventions [13]. In 2022, there were 92 countries providing at least one needle exchange service [4].

In Ireland, needle and syringe exchange programmes have been a key component of successive drug and alcohol policies, even before harm reduction was identified as a public health response [14,15]. As noted above, these were initially developed in the late 1980s as fixed-site services in areas that experienced high levels of substance use. Some areas developed mobile needle exchange services in response to the spread of injecting drug use beyond inner city Dublin regions. However, there remained large areas of the country with poor access to these services. Government strategy therefore recommended the expansion of the needle exchange programme to community pharmacy settings [14]. These provide free access to sterile injecting equipment from participating pharmacies throughout Ireland during normal trading hours. They also collect used equipment (although returned needles are not required to obtain clean needles) and refer patients for treatment, blood-borne virus testing and hepatitis B vaccinations. Community pharmacies have been established in a number of other countries to help improve the accessibility of needle exchange services [16]. By 2012, there were 71 pharmacies actively providing needle exchange services in Ireland [17], with the current strategy recommending further expansion of the programme in areas of need [15]. In 2022, there were 90 pharmacies actively providing needle exchange services.

International reviews of the impact of community pharmacy needle exchange services have shown that they have a positive effect in terms of reducing high-risk injecting behaviours such as needle-sharing, in addition to a range of other positive health outcomes [18]. However, in Ireland, there is limited up-to-date information on the impact of community pharmacy needle exchanges. For example, a review of all needle exchange programmes by Bingham et al. [19], based on 2012 data, highlighted data collection gaps and recommended a standardised reporting mechanism. Bates et al. [20] utilised 2013 data to show the positive impact of the Pharmacy Needle Exchange Programme in terms of uptake and referrals to other services. There have been no studies undertaken in Ireland since 2015. In addition, there is a relative absence of studies undertaken elsewhere that monitor patterns of use over time. This is important, as there is a need to determine usage patterns in the context of changing drug markets and user preferences. For example, there has been an increase in cocaine use [21] and treatment [22] in Ireland in recent years, with crack cocaine use emerging as a problem among marginalised groups [23]. Such information is crucial in terms of ensuring that harm reduction initiatives continue to meet the needs of PWID. The study aimed to determine patterns of needle distribution and return in community pharmacies in Ireland.

## 2. Materials and Methods

**Figure 1:**
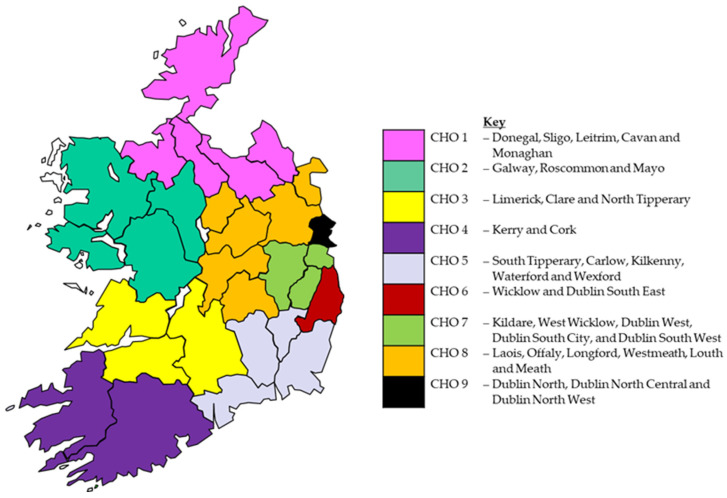
Community Health Organisation areas in Ireland (CHO).

The Health Service Executive (HSE) in Ireland collects Key Performance Indicators (KPIs) on a quarterly basis to monitor the performance of each division, set targets for ongoing service delivery, and develop strategic plans for forthcoming years. All community pharmacies that participate in the Community Pharmacy Needle Exchange Programme submit monthly KPI data to the HSE. Each participating pharmacy inputs anonymised data for each free transaction into an audit form or a database. No personal data or unique identifiers are collected from those using the service and registration is not required. Pharmacies receive a payment for participating in the scheme. Following a data validation process, monthly aggregated data are then submitted on a quarterly basis to the HSE Planning and Business Information Unit (PBI). The PBI then construct end-of-year totals for each KPI for all pharmacies at regional and national levels. The following data are collected: the number of participating pharmacies, the number of individuals attending the programme each month, the number of needle/syringe packs provided (three different types of packs containing needles, syringes, swabs, vials, citric acid packs, a personal sharps container, and water for one, three and ten injections), the number of needle/syringe packs returned, the percentage of needle/syringe packs returned, the number of clean needles provided each month (collected since 2017) and the average number of clean needles (and accompanying injecting paraphernalia) per individual each month. From these data, the KPIs from 2015 to 2022 for the programme were obtained from the PBI. Yearly totals of actual usage for each KPI from 2015 to 2022 were calculated for Ireland overall and were disaggregated by Community Health Organisation Area (CHO). These geographical areas are responsible for the delivery of primary and community services in Ireland (see Figure 1). Each CHO contains a mix of urban and rural locations. CHO6, CHO7 and CHO9 do not have any pharmacies included in the programme. These CHOs cover Dublin city and surrounding counties and have other needle exchange services, which were established prior to the introduction of the pharmacy service due to the large number of injecting drug users. The KPIs were compared to yearly targets that had been set for the service by the HSE. The HSE targets are shown in Table 1. The study utilised aggregated KPIs, which did not permit a detailed statistical analysis of patterns. The analysis focused on using counts, percentages, and means to describe patterns from 2015 to 2022. As the analysis was based on aggregate data, it was not deemed necessary to obtain ethical approval for the study. To facilitate the analysis of patterns, the mean of 2015 and 2017 was used to provide an estimate for 2016.

## 3. Results

### 3.1. Number of Pharmacies in Programme

**Figure 2:**
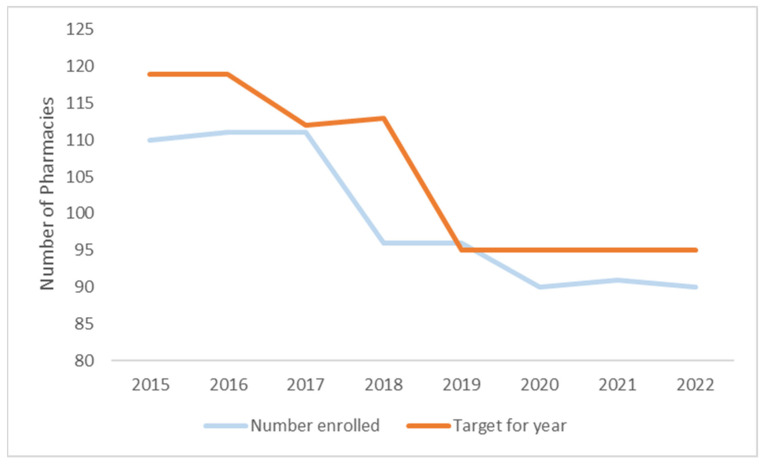
Number of pharmacies providing programme 2015–2022.

In 2022, there were 90 pharmacies in Ireland actively providing the Pharmacy Needle Exchange Programme. This is 5% lower than the KPI target set for 2022 (95 pharmacies). The number of pharmacies providing the programme has reduced by 18% since 2015 (Figure 2), with two sharp declines experienced in 2018 and in 2020. It is below the KPI target set for every year except 2019.

### 3.2. Number of People Using Programme

**Figure 3:**
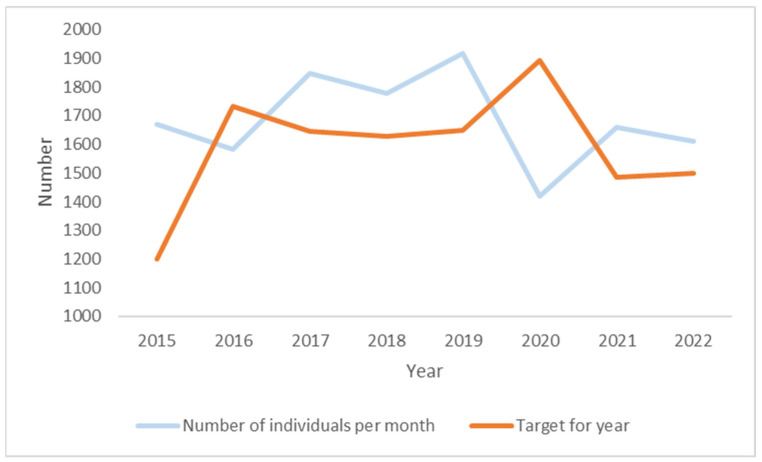
Number of unique individuals using programme 2015–2022.

In total, 1612 unique individuals per month used the Pharmacy Needle Exchange Programme in 2022 (mean = 17.9 people per pharmacy). This is 7.5% higher than the KPI target set for 2022 (1500 people). The number of unique individuals using the programme increased by 15% from 2015 to 2019, followed by a decline of 16% from 2019 to 2022. Overall, between 2015 and 2022, there has been a 3% decline in the number using the service (Figure 3). In terms of KPI targets, there is a fluctuating pattern, with targets met for five of the years (2015, 2017–2019, 2021), and not met for three of the years (2016, 2020, 2022) included in the analysis.

### 3.3. Packs Provided and Returned

**Figure 4:**
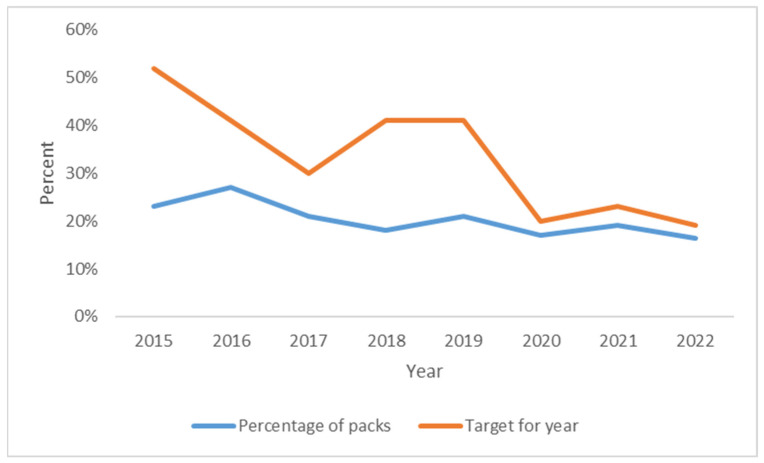
Proportion of packs returned (2015–2022) *. * 2016 = mean of 2015 plus 2017, as data are missing.

In 2022, 3775 packs per month (containing needles, syringes, swabs, vials, citric acid packs, sharps containers and water) were provided. This represents an overall decline of 19% compared to 2015. The overall proportion of packs returned has also declined, from 23% in 2015 to 16% in 2022 (Figure 4). This represents a 28% decline in the proportion of packs returned. Overall KPI targets have not been met in any of the years from 2015 to 2022.

### 3.4. Sterile Needles Provided

**Figure 5:**
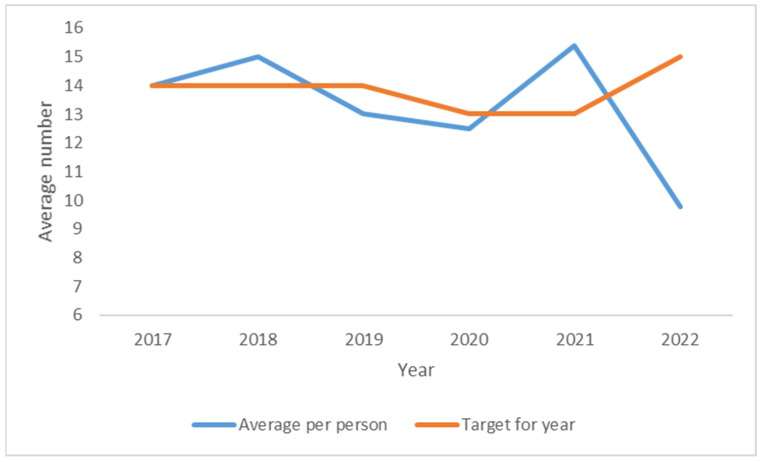
Average number of needles per individual per month (2015–2022).

In 2022, the programme provided 21,296 needles per month, with each person attending receiving 9.8 needles on average per month. Compared to 2017, the number of sterile needles provided has declined by 16%. There has been a 4.2 percentage point reduction in the average number of needles per individual since 2017 (Figure 5). KPI targets have not been met for half of the years included in the analysis (2017, 2018 and 2021).

### 3.5. Regional Comparsions

Table 2 compares KPIs by CHOs within Ireland. The greatest proportion of pharmacies providing the programme in 2022 were located in CHO8 (32%, *n* = 29), CHO4 (18%, *n* = 16), and CHO5 (16%, *n* = 14). All of the CHOs involved in the programme have experienced a decline in the number of pharmacies participating since 2015, with the largest decline experienced in CHO1 (25%; from 12 to 9), CHO5 (22%; from 18 to 14) and CHO3 (20%; from 15 to 12). In 2022, the greatest proportion of individuals using the programme were located in CHO4 (48%, *n* = 774), CHO5 (23%, *n* = 372) and CHO8 (20%, *n* = 328). CHO4 (48.4) and CHO5 (26.5) had the highest mean number of people attending per pharmacy in 2022. CHO4 has experienced an increase in people using the programme since 2015 (98%). The number of people using the programme in all other CHOs has declined, with the largest declines experienced in CHO3 (84%), CHO2 (53%) and CHO8 (27%). In terms of returned packs, the CHOs with the largest proportion returned were CHO8 (33%), CHO5 (21%) and CHO1 (15%). With the exception of CHO8 (where the proportion returned increased by 33%), all CHOs have experienced a decline in the proportion of packs returned (23–70%). The mean number of needles per individual was greatest in CHO1 (13.4), CHO5 (11.9) and CHO4 (11.4). Since 2015, the mean number of clean needles per individual has declined in all CHOs, with CHO1 and CHO2 experiencing the largest decline (72% and 70%, respectively).

## 4. Discussion

Community pharmacy needle exchange services are a key component of Ireland’s public health harm reduction approach to reduce the negative impact of injecting drug use. By monitoring usage patterns, the study provides an insight into whether they are meeting the needs of PWID. Overall, the services have experienced a decline in the numbers attending and, in many instances, have not met the targets that were set. The emerging patterns raise a number of issues in terms of policy implications and provide an insight into the provision of harm reduction services in the context of changing drug markets in Ireland.

### 4.1. Size and Use of Service

Between 2015 and 2022, there has been a decline in the number of pharmacies providing (18%), and the number of people (3%) using, the programme. This has coincided with a 19% decline in the number of packs provided and a 16% reduction in the number of sterile needles provided (since 2017).

The declining patterns of use are of concern, since reduced investment in harm reduction initiatives such as needle exchange services has been identified as a factor contributing to HIV outbreaks in Ireland and a number of other countries [24]. Indeed, the closure of the largest needle exchange in Glasgow, Scotland, has been identified as a significant setback in terms of preventing the spread of HIV among PWID [25].

Participation by pharmacies in the programme in Ireland is voluntary, which means that they can choose to withdraw, leaving gaps in service provision. Broad coverage has been identified as a key element of effective needle and syringe provision [26]. It is important that gaps in service provision are filled, for example by encouraging pharmacies to provide services in areas without a service, or by expanding existing community drug treatment services.

The declining patterns in terms of size and usage also raises the question of whether there has been a change in drug markets in Ireland, with fewer PWID presenting for treatment. Since 2015, there has been a gradual decline in estimates of problematic opioid use in Ireland [27], but this decline has not been significant. During this period there has been an increase in the number of non-fatal overdoses discharged from hospital, with opioids involved in over two thirds of cases [28]. In 2020, there were 409 deaths due to poisoning, with 70% involving opioids [29]. This represents an 8% increase compared to 2017. Since 2015, there has been an increase in the number of people accessing Opioid Agonist Treatment (OAT) and this was particularly apparent during the COVID-19 pandemic, when individuals were fast-tracked into treatment [30]. These patterns suggests that the demand for sterile needles should not have significantly changed during this period.

Declining patterns may be due to some users choosing to obtain injecting equipment elsewhere, such as travelling to the larger needle exchange programmes in Dublin where there would be greater anonymity. The decline in the number of sterile needles provided helps to demonstrate this issue. In England, online purchasing of needles has been suggested as a factor to explain declining utilisation since COVID-19 [31]. In the current study, the number of individuals using the service did decline in 2020, coinciding with COVID-19, which does suggest that lockdown restrictions may have played a role in declining patterns of usage. During COVID-19, a home delivery needle and syringe service was established [32], along with increased provision of OAT services [33], which may also have meant that fewer people would have needed to attend a needle exchange. Since 2020, patterns have only recovered to 2015 levels, which suggests that COVID-19 may have had an enduring impact on behaviour patterns. In England, the reuse and sharing of equipment has been suggested as a factor to explain the continued decline in people using the service since COVID-19 [31].

Heroin users can inject multiple times a day and are at high risk of reusing or sharing needles due to cravings if they do not have access to an adequate supply of sterile injecting equipment [34]. It has been estimated that a heroin user needs to inject 2.8 times a day [35], which equates to 85 times a month. This is over eight times higher than the number of sterile needles provided per individual using the Pharmacy Needle Exchange Programme in 2022. Although the proportion of active heroin injectors that use the programme is not known, this does nevertheless raise concerns that some individuals are reusing their equipment, and the core messages of the harm reduction advice may need to be reemphasised. Further research could be conducted with this cohort to identify the actual level of injecting and whether there are many situations where people alternate between injecting and smoking.

The reasons behind this declining pattern of usage are unclear and warrant further investigation. In a systematic review of 36 studies, Gionfriddo et al. [36] found that perceptions of safety, the potential for improper disposal of syringes, and concerns about other customers are key barriers that discourage pharmacists from selling clean needles to PWID.

Recent prevalence studies have highlighted the increase in cocaine use in Ireland and identified the emergence of ‘crack’ cocaine use among a marginalised population that may previously have used heroin [23]. Cocaine use can cause intense craving [37], with users also experiencing irritability and anxiety [38]. There is a need to investigate whether such behavioural issues are discouraging pharmacies from engaging with the needle exchange service. All pharmacies should have protocols in place to ensure the safety of their employees. PWID may present with challenging behaviour, and staff must possess the necessary skills and have the supporting infrastructure to deal with such challenging behaviour. A review by Lawson et al. [39] highlighted stigma and the perception of negative consequences as barriers to accessing sterile injecting equipment.

The withdrawal of pharmacies from the scheme in the current study may have made the service less accessible to PWID, who, as a result, choose more accessible sources of equipment. They may also have additional needs that may not be met by the Pharmacy Needle Exchange Programme. For example, there is evidence that suggests that crack cocaine use is increasing among PWID in Ireland. The EMCDDA have reported this as an emerging pattern in Portuguese drug consumption rooms [3] and it has also been reported in Scotland [40]. If this is the case, then additional harm reduction initiatives may be required (such as the provision of sterile crack pipes). Such emerging drug use patterns may require additional training for pharmacies, as the needs of IDUs who also use crack cocaine may present additional challenges.

### 4.2. Packs Returned

Discarded needles and used equipment that was used to inject drugs can increase the risk of injury and the spread of infectious diseases such as hepatitis C and HIV [41]. Although these risks are relatively low [42,43] (with transmission risk in Ireland also reducing due to a decline in HIV incidence rates [44,45]), they nevertheless represent a health and safety hazard, causing negative perceptions and anxiety among communities, particularly among parents of young children [42,46]. Such discarded injecting paraphernalia can lead to communities opposing the development of services in an area, so it is important that this be addressed in a proactive manner.

The Pharmacy Needle Exchange Programme aims to minimize this impact by encouraging the return of used items from the packs that it provides, particularly needles [19]. Although failing to return needles may not mean that they were disposed of unsafely, it is nevertheless of concern that, in 2022, only 16% of packs were returned, which represents a 21% decline compared to 2015. A study of the programme using 2012 data found that 39% of packs were returned [17], which shows that rates for 2015–2022 are considerably lower than those achieved in 2012. Low return rates were also highlighted in a review of the service by Bates et al. [20]. Data for Northern Ireland needle and syringe exchange services for 2019–2020 show that 31% of Cin-Bins (container for safe disposal of needles which is included in every pack) were returned [47]. A review (although somewhat dated) of 26 studies of needle exchange programmes reported an overall return rate of 90% [48]. Unpublished data from needle exchange services in Mandura in Western Australia show an overall return rate of 94% from 2021 to date.

These studies suggest that high return rates can be achieved, but barriers in Ireland may need to be overcome. The Centre for Disease Control and Prevention (CDC) in reviewing studies state that PWID may not return needles to exchange programmes due to concerns about being arrested for the possession of syringes, a lack of sharps containers, needles taken by other PWID due to low supply and high demand, homelessness and living conditions [49].

In some areas in Ireland, needle disposal bins have been installed in areas identified as having a problem with drug-related waste that may be more accessible to PWID [50]. Although the extent of this initiative is not known, it might be that needles and used items provided by the Pharmacy Needle Exchange Programme are returned to these disposal bins. There are no distinguishing identifiers on products obtained from the Pharmacy Needle Exchange Programme. Users of the programme have to enter the pharmacy and put used equipment (in a sharps container) into secure bins. This may deter returns if users perceive this process to be inconvenient. In addition, if they feel stigmatised for being a drug user, they may try to minimise the number of times they use such facilities. For example, Paquette et al. [51] report the significant impact stigma has on syringe access in USA, particularly pharmacies. This issue is also highlighted in a review by Tung et al. [46]. Tung et al. [46] also outline a number of initiatives, such as the use of sharps containers that remove the needle barrel from the needle (potentially preventing crimination), drop boxes and one-way disposable bins in a wider range of locations, the use of vending machines to dispense needles, mail slots in bathrooms and disposal bins designed to look like post boxes (to be less conspicuous). In Mandura in Western Australia, one of the stipulations that contributes to their 94% return rate is the supply of free needles only upon the return of used items [52]. Alternative systems of service delivery do need to be considered to improve return rates. In addition, the degree to which the return of equipment is emphasized by pharmacies is not known. It might be that, due to the lowering rates of hepatitis C and HIV among IDUs [44,45], there may be a lack of emphasis of the importance of returning used equipment.

Complacency in relation to blood-borne virus (BBV) transmission among both staff and service users may have contributed to the low return rate. A previous study of the programme emphasised the need for service providers to advise users about the safe disposal of used needles and equipment [20]. Although a standardised leaflet is provided to users [53], this does not contain information on the return of packs and there appears to be a lack of standardised procedures in place in terms of facilitating and encouraging users to return packs. Such procedures, if accompanied by training, may help promote the return of used equipment.

### 4.3. Regional Comparisons

In examining the programme by health regions within Ireland (CHO), it can be seen that patterns do differ by area. For example, while CHO8 has the largest proportion of pharmacies (32%), CHO4 has the greatest proportion of individuals using the programme (48%). On average, there are 48 people using each pharmacy in CHO4, which is almost double that of CHO5 (*n* = 27) and four times that of CHO8 (*n* = 11). The number of pharmacies in the programme has declined in all CHOs since 2015. Although there may be a rationale to reduce the number of pharmacies providing the service in CHOs with low take-up of the service, for CHOs with large numbers of users (e.g., CHO4), there may be a need to consider expanding the service to more pharmacies. This approach could spread the burden on existing pharmacies that provide the service, and may help to reduce the distance for people to travel (particularly in rural areas). Expansion plans would also need to investigate issues such as the recruitment of pharmacies, to help ensure that sufficient numbers of pharmacies apply to provide the service. A concerning finding from regional comparisons is the variation in the proportion of packs returned by CHO (6–33%) which would warrant further investigation to identify variations in programme implementation at a regional level that may be used to explain and rectify the low proportion of returns.

### 4.4. Comparisons with Other Countries

Although the decline in usage of the service suggests scope for improvement, comparisons with other countries show that, in terms of usage, data for the Pharmacy Needle Exchange Programme are broadly comparable, and indeed better than many countries. A review by Colledge-Frisby et al. [54] showed that the average number of clean needles per individual for countries in western Europe was 115.3 per year, which compares to 117.6 for the Pharmacy Needle Exchange Programme. Using the WHO criteria employed by this review (‘low’, ‘moderate’, ‘high’), the level of provision for the programme would be classified as ‘moderate’. In addition, the European Drug Report 2023 [3] shows that Ireland ranks 15th out of 27 countries in terms of the number of syringes distributed through specialised programmes (although this does not control for population size or prevalence levels). Thus, while improvements can be made, the programme KPIs for needles are broadly favourable when compared to other countries.

### 4.5. Study Limitations

The study is limited in that it focuses on a series of indicators that have been collected for the service. There was no interaction or feedback collated from either staff or service users. The indicators are broad and do not provide detailed information in terms of the operation of the programme, individual usage patterns (as the data are anonymous with no unique identifiers), nor the injecting behaviour of PWID (such as whether needles are shared). They may not accurately reflect the ever-evolving patterns of drug use, and as such, their usefulness in terms of developing and planning the service may have diminished over time. Ksobiech [55] has noted that, to improve needle exchange programmes, there is a need to record more than ‘the basics’, such as needles distributed and returned. In addition, the use of aggregate data meant that a detailed statistical analysis could not be undertaken. Such analysis would have been beneficial in terms of providing insights into the key predictors of patterns. Comparisons of KPIs for existing fixed-site services in Ireland were not made due to an absence of published studies and data unavailability at the time of the analysis.

Although the current indicators provide a valuable insight into access and patterns of use over time, they nevertheless need to be reviewed to determine if additional information should be routinely collected to monitor the performance of the programme. There is a need for the Pharmacy Needle Exchange Programme to adapt to changing drug trends in an agile manner to ensure that those who are attending the service are receiving appropriate interventions (e.g., consideration of sterile pipes for ‘crack’ cocaine).

In addition, to provide a better understanding of the reasons that may explain the patterns, the study would have benefitted from input from both service providers and service users. Detailed reviews have previously been undertaken that utilised such input [19,20], and it is suggested that an up-to-date detailed review is now required to complement the indicators that were utilised in the current study.

## 5. Conclusions

The declining patterns of usage of the Pharmacy Needle Exchange Programme has raised a number of important issues for consideration. It is hoped that this will help ensure that the needs of those that use it are better met in the future. Despite the declining patterns of usage, it is worth noting that, in 2022, it provided 21,296 sterile needles per month in areas of Ireland that are not served by fixed-site services. As such, the service can be viewed as a significant harm reduction initiative and remains a key component of Ireland’s harm reduction approach to drug use [15]. Through regular review and monitoring, it is anticipated that this service can significantly enhance its harm reduction impact in the future. This review was conducted eight years after reviews undertaken by Bingham et al. [19] and Bates et al. [20]. Given the issues that have been identified and the changing drug trends in Ireland, it would be prudent to conduct such reviews on a more regular basis, perhaps every 2–3 years.

## Figures and Tables

**Table 1:** KPI Targets for the Community Pharmacy Needle Exchange Programme 2015–2022.

KPIs	Year							
	2015	2016	2017	2018	2019	2020	2021	2022
Number of pharmacies recruited	119	119	112	113	95	95	95	95
Number of individuals per month	1200	1731	1647	1628	1650	1894	1486	1500
Percentage of needle/syringe packs returned	52	41	30	41	41	20	23	19
Average number of clean needles per individual per month	14	14	14	13	13	15	14	14

**Table 2:** KPIs for the community pharmacy needle exchange programme by community health organisation area (CHO).

KPIs	Community Health Organisation Area (CHO) *					
	CHO1	CHO2	CHO3	CHO4	CHO5	CHO8
Proportional breakdown of pharmacies providing programme 2022	10%	11%	13%	18%	16%	32%
Proportional breakdown of individuals using programme 2022	2%	4%	3%	48%	23%	20%
Percentage change in pharmacies providing programme 2015–2022	−25%	−17%	−20%	−16%	−22%	−15%
Percentage change in individuals using the programme 2015–2022	−20%	−53%	−84%	98%	−3%	−27%
Mean number of individuals using programme per pharmacy 2022	3.5	6.4	3.6	48.4	26.5	11.3
Proportion of packs returned 2022	15%	6%	7%	8%	21%	33%
Percentage change in the proportion of packs returned per month 2015–2022	−70%	−66%	−67%	−52%	−23%	33%
Mean number of needles provided per individual per month 2022	13.4	2.4	9.2	11.4	11.9	10.4
Percentage change in mean number of needles provided per individual per month 2015–2022	−72%	−70%	−34%	−5%	−20%	−31%

* CHO1—Donegal, Sligo, Leitrim, Cavan and Monaghan; CHO2—Galway, Roscommon and Mayo; CHO3—Limerick, Clare and North Tipperary; CHO4—Kerry and Cork; CHO5—South Tipperary, Carlow, Kilkenny, Waterford and Wexford; CHO8—Laois, Offaly, Longford, Westmeath, Louth and Meath.

## Data Availability

Availability of data and material database available from National Social Inclusion Office, Health Service Executive, Ireland.
